# Refractory response to entrectinib for ROS‐1 rearranged NSCLC with concurrent de novo TP53 mutation showing good response to CNS lesion, but poor duration of response: A case report

**DOI:** 10.1111/1759-7714.15044

**Published:** 2023-08-06

**Authors:** Kentaro Ito, Miho Nishio, Kentaro Fujiwara, Yoichi Nishii, Kengo Ushiro, Hiroki Yasui, Osamu Hataji

**Affiliations:** ^1^ Respiratory Center Matsusaka Municipal Hospital Matsusaka Japan; ^2^ Department of Biostatistics Yokohama City University of Medicine Yokohama Japan; ^3^ Department of Clinical Laboratory Matsusaka Municipal Hospital Matsusaka Japan

**Keywords:** brain metastasis, entrectinib, ROS‐1 fusion, TP53 mutation

## Abstract

Entrectinib, a ROS‐1 inhibitor, has been shown to be effective for patients with ROS‐1 fused NSCLC, and has been established as the standard of care for this population. Entrectinib has been shown to achieve a better response to brain metastasis due to the characteristic of the drug having a weak interaction with P‐glycoprotein and, even in prospective studies, the intracranial response is higher. Patients have been known to acquire resistance to molecularly targeted drugs such as EGF‐TKIs or ALK‐TKIs during targeted therapy. Similarly, the mechanisms of resistance to entrectinib have been reported, but information about the effects of TP53 mutation with entrectinib are still limited. Here, we experienced a case of a patient with ROS‐1 fusion and concurrent TP53 mutation who was treated with entrectinib, resulting in a response to brain metastasis but rapid resistance to entrectinib. Our case demonstrates both the intracranial activity of entrectinib and the potential for resistance to entrectinib due to TP53 mutation.

## Graphical abstract

Entrectinib showed a transient response to brain metastases but rapid disease progression at 4.7 months in the patient with NSCLC harboring ROS‐1 fusion and TP53 mutation. This case suggests that entrectinib, like other molecularly targeted drugs, may be resistant to TP53 mutations, which is the first report of entrectinib for TP53 mutation. As previously reported for TP53 mutations with EGFR TKIs, entrectinib for ROS1 mutations also showed a transient therapeutic effect in patients with TP53 mutations, suggesting the possibility of early resistance in these patients.

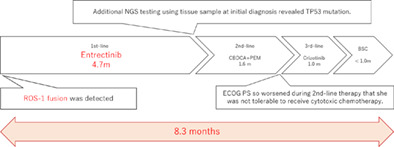

## INTRODUCTION

In the current treatment of non‐small cell lung cancer (NSCLC), genetic mutation testing is essential for patients with driver mutations, as molecularly targeted drugs targeting these mutations show dramatic efficacy. ROS‐1 rearrangement is one of the driver oncogenes in lung cancer, detected in ~1%–2% of NSCLC,^1^ and ROS‐1 inhibitors, crizotinib and entrectinib, are approved for the treatment of ROS‐1 rearranged NSCLC. Crizotinib demonstrated an objective response rate (ORR) of 72% and median progression‐free survival (PFS) of 19.3 months for ROS‐1 rearranged NSCLC in the PROFILE 1001 trial.^2^, ^3^ Entrectinib showed an ORR of 67.9% and a median PFS of 15.7 months for ROS‐1 rearranged NSCLC in an integrated analysis of phase 1 and 2 studies.^4^ Entrectinib was reported to prolong central nervous system (CNS) exposure due to a weak interaction with P‐glycoprotein, which is a major efflux transporter of the blood–brain barrier.^5^ Intracranial response to crizotinib was not assessed in the PROFILE 1001 trial, whereas the integrated analysis of the entrectinib trials showed an intracranial response rate of 80% (95% CI: 59.3–93.2).^4^ Patients administered molecularly targeted drugs, such as epidermal growth factor receptor‐tyrosine kinase inhibitors (EGFR‐TKIs) or anaplastic lymphoma kinase (ALK)‐tyrosine kinase inhibitors (TKIs), have been shown to develop resistance during treatment. The mechanisms of resistance to crizotinib and entrectinib have been reported in ROS‐1 rearranged NSCLC; however, data regarding the resistance to entrectinib is limited. Here, we report a case of a patient with ROS‐1 rearranged NSCLC harboring the TP53 mutation who experienced early disease progression on entrectinib therapy, despite a dramatic response to the intracranial lesion.

## CASE REPORT

**FIGURE 1:**
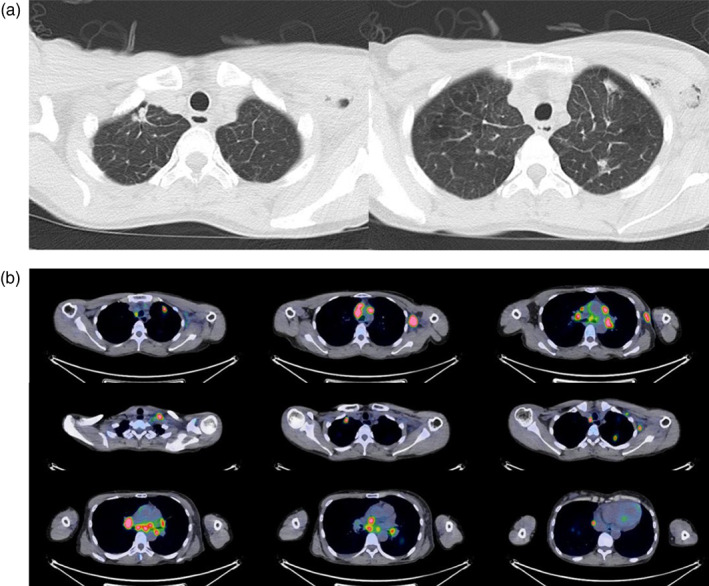
Imaging at initial diagnosis by (a) chest computed tomography (CT) and (b) positron‐emission tomography (PET)‐CT.

**FIGURE 2:**
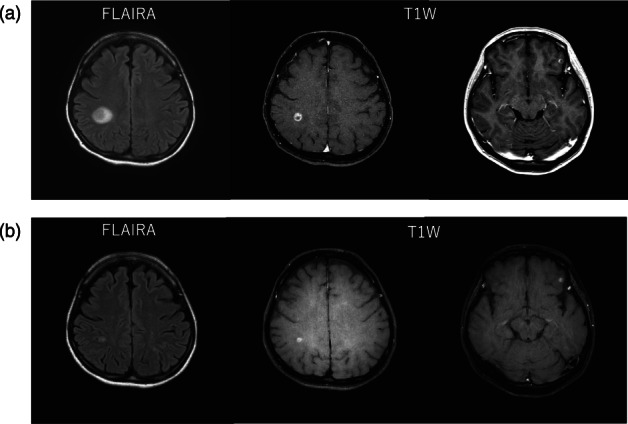
Head magnetic resonance imaging (MRI) (a) at initial diagnosis, and (b) 21 days after entrectinib administration.

A 45‐year‐old woman noticed enlarged lymph nodes in the left axilla, which had been gradually worsening for several months. She had no previous medical history and, to her knowledge, no family history of cancer. She consulted a local physician who suspected a malignant lymphoma and she was subsequently referred to the hematology department of a general hospital. A computed tomography (CT) scan showed multiple lymphadenopathies, cancer lymphangiopathy, and brain metastasis. Breast echography and mammography were also performed, but no breast cancer lesions were detected. Needle biopsy of the left axillary lymph node was performed to diagnose the malignant lymphoma, which led to the diagnosis of lung cancer metastasis. The pathological diagnosis was lung adenocarcinoma, TTF‐1 positive, napsin A positive, p40 negative, and CK5/6 negative. Blood tests for tumor markers showed an elevated CEA level of 14.3 ng/mL. Positron emission tomography‐CT (PET‐CT) scan showed multiple metastases with accumulations in the left cervical lymph node (LN), left supraclavicular fossa LN, mediastinal LN, right upper paratracheal LN, thoracic para‐aortic LN, subtracheal LN, right hilar LN, left hilar LN, right pericardial LN, left brachial LN, right pulmonary apex, left upper lobe node, and right supradiaphragmatic node (Figure 1). Head magnetic resonance imaging (MRI) also confirmed two metastases in the right parietal lobe and one metastasis in the left parietal lobe (Figure 2a). Gene testing by Oncomine DxTT using a tissue biopsy sample from the lymph node metastasis revealed ROS‐1 rearrangement. Based on the results of these tests, she was diagnosed with advanced (c‐Stage IVB) ROS‐1 rearranged lung adenocarcinoma with brain metastases, and treatment with entrectinib was decided upon. The following points were taken into consideration when deciding against prior radiotherapy of the brain metastases: (1) If gamma knife surgery is performed, it takes time because the treatment is performed at another facility. (2) Brain metastases are wait‐and‐see due to size, location, and lack of neurological symptoms. (3) When local treatment of brain metastases is prioritized, other site lesions, including lung lesions and lymph node metastases, are left untreated during this period of radiotherapy. (4) Current reports indicate that the response to entrectinib in brain metastases is relatively good. For these reasons, she started entrectinib therapy without prior radiotherapy for brain metastases, and radiotherapy would be reconsidered after early imaging evaluation. On day 20 after entrectinib administration, the brain metastases were evaluated by head MRI to determine whether to add radiotherapy, and the results showed that the treatment was effective (Figure 2b).

**FIGURE 3:**
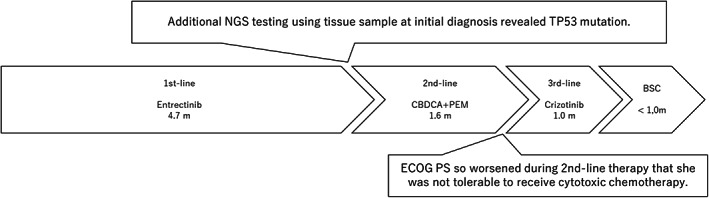
Clinical course.

CT imaging also showed an improvement of the lung lesions at the time of assessment. However, on day 143 (~4.7 months) of entrectinib therapy, the lung lesions showed disease progression with lymphadenopathy. Suspecting that the ROS‐1 rearrangement result by Oncomine DxTT was pseudo‐positive, we performed additional next‐generation sequencing (NGS) testing using the Oncomine comprehensive assay v3, which detected CD74‐ROS1 fusion, and concurrent TP53 mutation (Table 1). We proposed that second‐line therapy including combination therapy with an immune checkpoint inhibitor, such as the regimen in IMpower150 or KEYNOTE189 based on the results of PD‐L1 high expression with over 95% was initiated, but after explaining to the patient and her family about immunotherapy for ROS‐1 rearranged NSCLC and the potential adverse events due to immunotherapy, they decided that she should not receive immunotherapy taking into consideration her condition. We exchanged chemotherapy with platinum‐based pemetrexed, but the efficacy was limited with duration of response (DoR) of 1.6 months, and Eastern Clinical Oncology Group performance status (ECOG PS) worsened due to disease progression. For poor ECOG PS, TKI therapy is the only tolerable treatment for patients. In addition, the patient refused the cytotoxic agent chemotherapy and immunotherapy, and therefore received crizotinib therapy. The subsequent therapy of crizotinib, however, was also less effective with DoR of 1.0 months. The patient died with 8.3 months of overall survival from the administration of entrectinib as first‐line therapy (Figure 3).

**TABLE 1:** The results of gene profiling by oncomine comprehensive assay v3.

Gene	Mutation	Read count (RC)/allele frequency (AF)
CD74‐ROS1	Fusion	RC: 27536
TP53	R273	AF: 32.5%

## DISCUSSION

This case demonstrated two clinical features; early response of entrectinib for radiotherapy naïve CNS lesions, and nonsustained response of entrectinib with concurrent TP53 mutation. To our best knowledge, this is the first report of a case treated with entrectinib for ROS1 fusion with concurrent TP53 mutation.

First, this case showed a good response of entrectinib for brain metastases at first assessment, even without prior radiotherapy. Recently a multi‐institutional retrospective study for patients with *EGFR*‐mutated or ALK‐rearranged NSCLC evaluated the survival benefit between patients who received CNS‐penetrant TKI therapy alone compared with those who received radiotherapy for brain metastases prior to TKI therapy.^6^ This report showed that there was no difference in clinical outcomes when a CNS‐penetrant TKI was used in advance, regardless of prior radiotherapy for brain metastases. Although the CNS‐penetrant TKIs in this report did not include entrectinib, previous reports showed sufficient data on the response to entrectinib in brain metastases to identify entrectinib as one of the CNS‐penetrant TKIs. In an integrated analysis of phase 1 and phase 2 entrectinib treatment, the intracranial ORR was reported to be 80% (95% CI: 59.3–93.2).^4^ As described previously, basic medical science suggests that entrectinib will continue to be exposed intracranially due to the weakness of interaction with P‐glycoprotein.^5^ Intracranial response to crizotinib in ROS‐1 rearranged NSCLC has not been reported, and the penetration of crizotinib is likely not good according to the results of the CROWN trial, a phase 3 trial comparing crizotinib and lorlatinib in patients with ALK‐rearranged NSCLC, which has shown that the intracranial response to crizotinib in patients with ALK‐rearranged NSCLC was 23% (95% CI: 5–54).^7^

Second, this case showed good response to entrectinib for brain metastasis, although the response was not durable. The mechanisms of resistance to crizotinib and entrectinib for ROS‐1 rearranged NSCLC have been reported; however, TP53 mutation has not been listed.^8^, ^9^ To the best of our knowledge, this is the first case report with ROS‐1 rearranged NSCLC who experienced a refractory response to entrectinib, with concomitant mutation of TP53. TP53 mutation is already well‐known as a mechanism of resistance to molecular targeted therapy for other oncogenes such as EGFR and ALK, and several reports indicated that crizotinib and brigatinib have shown less efficacy for NSCLC with ALK‐rearrangement concurrent with TP53 mutation.^10^, ^11^ Lorlatinib has also been shown to have decreased PFS in TP53‐mutated ALK or ROS‐1 positive NSCLC.^11^ Vokes et al. reported that EGFR‐TKIs showed similar response rates between patients with the TP53 mutation and TP53 wild‐type; however, there was a difference in the duration of response between the two groups, concluding that TP53 mutation facilitates resistance to EGFR‐TKIs resulting in shorter PFS.^12^ Although the report was for EGFR‐TKIs, their conclusions are consistent with our case, which also showed a transient response to brain metastasis without continued response. From our case, we hypothesize that a similar phenomenon is found with entrectinib for NSCLC with ROS‐1 fusion and TP53 mutation. TP53 mutation is also one of the poor prognostic factors, and an unfavorable predictive factor for chemotherapy in ALK‐rearranged NSCLC.^13^ The duration of response to chemotherapy in this case was also only 1.6 months, which is consistent with previous reports.

In this case, additional NGS testing using OCA v3 detected not only CD74‐ROS1 fusion but also concurrent TP53 mutation. TP53 mutation is not among the detectable variants of the Oncomine DxTT, which was used at initial diagnosis, while OCA v3 testing can detect 161 genes including TP53 mutation. As previously described, TP53 mutation is a poor prognostic factor, and an unfavorable predictive factor for TKI therapy, probably even for ROS1 inhibitors. Our case with TP53 mutation showed transient response followed by early progression, therefore we suggest from this case that close imaging follow‐up is necessary in cases with TP53 mutation.

In cases with TP53 mutation, even if detected, chemotherapy also has a reduced therapeutic effect at this time, and an effective treatment option for TP53 mutation has not yet been established. In EGFR‐positive lung cancer, the RELAY trial reported that the combination of ramucirumab and erlotinib was more synergistic in patients with TP53 mutations.^14^, ^15^ For the rationale of the additional effect of ramucirumab for TP53 mutated cases, data have been reported about the association of TP53 and the VEGF pathway. TP53 mutation status has been reported to be an independent predictor of VEGF‐A expression in a several reports,^16^, ^17^ while another report has suggested that TP53 mutations bind to the initiation site of the VEGFR2 promoter region and promote transcription of the VEGFR2 gene, resulting in increased VEGFR2 expression.^18^ These data suggest that combination therapy with anti‐VEGF antibodies may be effective against TP53 mutated cancers. Further investigation of novel treatment, including combination therapy with anti‐VEGF antibodies, is warranted to overcome the refractory response due to TP53 mutation in ROS1 rearranged NSCLC in the future. Although immunotherapy including combination therapy was an option for this case, the patient and her family decided not to go ahead with immunotherapy due to the insufficient immunotherapy data for ROS‐1 rearranged NSCLC, and the data from IMMUNOTARGET registry which showed that the progressive disease rate was worst with over 80% among the driver oncogene in the report,^19^ together with the concern about adverse events associated with immunotherapy. We hope that immunotherapy for ROS1‐rearranged lung cancer will continue to be validated by future data.

In conclusion, our case of an adenocarcinoma patient with ROS‐1 fusion concomitant with TP53 mutation which showed a transient response, with rapid response to brain metastasis but early systemic progression, suggests that assessment of efficacy of entrectinib to TP53 mutation should be frequent and should not be by response rate, but instead by duration of response.

## AUTHOR CONTRIBUTIONS

Kentaro Ito: Conceptualization, methodology, investigation, and writing original draft. Miho Nishio, Kengo Ushiro, Kentaro Fujiwara, Yoichi Nishii, and Tadashi Sakaguchi: Investigation. Osamu Hataji: Supervision.

## CONFLICT OF INTEREST STATEMENT

Kentaro Ito received a personal fee from Eli Lilly, Pfizer, Takeda Pharmaceutical, Chugai Pharmaceutical, AstraZeneca, Boehringer Ingelheim, Daiichi Sankyo, Ono Pharmaceutical, MSD, Amgen, and Taiho Pharmaceutical. Osamu Hataji received a personal fee from Eli Lilly, AstraZeneca, Takeda Pharmaceutical, Daiichi Sankyo, Sanofi, GlaxoSmithKline; received research fund from GlaxoSmithKline, AstraZeneca, and Sanofi. Miho Nishio, Kentaro Fujiwara, Yoichi Nishii, Kengo Ushiro, Tadashi Sakaguchi, and Hiroki Yasui have no relevant COI.

## References

[1] Bergethon K , Shaw AT , Ou SH , et al. ROS1 rearrangements define a unique molecular class of lung cancers. J Clin Oncol. 2012;30:863–870. 22215748 10.1200/JCO.2011.35.6345 PMC3295572

[2] Shaw AT , Ou SH , Bang YJ , et al. Crizotinib in ROS1‐rearranged non‐small‐cell lung cancer. N Engl J Med. 2014;371:1963–1971. 25264305 10.1056/NEJMoa1406766 PMC4264527

[3] Shaw AT , Riely GJ , Bang YJ , Kim DW , Camidge DR , Solomon BJ , et al. Crizotinib in ROS1‐rearranged advanced non‐small‐cell lung cancer (NSCLC): updated results, including overall survival, from PROFILE 1001. Ann Oncol. 2019;30:1121–1126. 30980071 10.1093/annonc/mdz131 PMC6637370

[4] Drilon A , Chiu CH , Fan Y , Cho BC , Lu S , Ahn MJ , et al. Long‐term efficacy and safety of entrectinib in ROS1 fusion‐positive NSCLC. JTO Clin Res Rep. 2022;3:100332. 35663414 10.1016/j.jtocrr.2022.100332 PMC9160474

[5] Fischer H , Ullah M , de la Cruz CC , Hunsaker T , Senn C , Wirz T , et al. Entrectinib, a TRK/ROS1 inhibitor with anti‐CNS tumor activity: differentiation from other inhibitors in its class due to weak interaction with P‐glycoprotein. Neuro Oncol. 2020;22:819–829. 32383735 10.1093/neuonc/noaa052 PMC7283026

[6] Thomas NJ , Myall NJ , Sun F , Patil T , Mushtaq R , Yu C , et al. Brain metastases in EGFR‐ and ALK‐positive NSCLC: outcomes of central nervous system‐penetrant tyrosine kinase inhibitors alone versus in combination with radiation. J Thorac Oncol. 2022;17:116–129. 34455066 10.1016/j.jtho.2021.08.009

[7] Shaw AT , Bauer TM , de Marinis F , Felip E , Goto Y , Liu G , et al. First‐line Lorlatinib or Crizotinib in advanced ALK‐positive lung cancer. N Engl J Med. 2020;383:2018–2029. 33207094 10.1056/NEJMoa2027187

[8] Lin JJ , Choudhury NJ , Yoda S , Zhu VW , Johnson TW , Sakhtemani R , et al. Spectrum of mechanisms of resistance to Crizotinib and Lorlatinib in ROS1 fusion‐positive lung cancer. Clin Cancer Res. 2021;27:2899–2909. 33685866 10.1158/1078-0432.CCR-21-0032 PMC8127383

[9] Ku BM , Bae YH , Lee KY , Sun JM , Lee SH , Ahn JS , et al. Entrectinib resistance mechanisms in ROS1‐rearranged non‐small cell lung cancer. Invest New Drugs. 2020;38:360–368. 31124056 10.1007/s10637-019-00795-3 PMC7066105

[10] Liu C , Liu C , Liao J , Yin JC , Wu X , Zhao X , et al. Genetic correlation of crizotinib efficacy and resistance in ALK‐ rearranged non‐small‐cell lung cancer. Lung Cancer. 2022;171:18–25. 35870258 10.1016/j.lungcan.2022.07.011

[11] Frost N , Christopoulos P , Kauffmann‐Guerrero D , Stratmann J , Riedel R , Schaefer M , et al. Lorlatinib in pretreated ALK‐ or ROS1‐positive lung cancer and impact of TP53 co‐mutations: results from the German early access program. Ther Adv Med Oncol. 2021;13:1758835920980558. 33613692 10.1177/1758835920980558 PMC7876585

[12] Vokes NI , Chambers E , Nguyen T , Coolidge A , Lydon CA , le X , et al. Concurrent TP53 mutations facilitate resistance evolution in EGFR‐mutant lung adenocarcinoma. J Thorac Oncol. 2022;17:779–792. 35331964 10.1016/j.jtho.2022.02.011 PMC10478031

[13] Kron A , Alidousty C , Scheffler M , Merkelbach‐Bruse S , Seidel D , Riedel R , et al. Impact of TP53 mutation status on systemic treatment outcome in ALK‐rearranged non‐small‐cell lung cancer. Ann Oncol. 2018;29:2068–2075. 30165392 10.1093/annonc/mdy333 PMC6225899

[14] Nakagawa K , Nadal E , Garon EB , Nishio M , Seto T , Yamamoto N , et al. RELAY subgroup analyses by EGFR Ex19del and Ex21L858R mutations for Ramucirumab plus Erlotinib in metastatic non‐small cell lung cancer. Clin Cancer Res. 2021;27:5258–5271. 34301751 10.1158/1078-0432.CCR-21-0273 PMC9662911

[15] Nishio M , Paz‐Ares L , Reck M , Nakagawa K , Garon EB , Popat S , et al. RELAY, Ramucirumab plus Erlotinib (RAM+ERL) in untreated metastatic EGFR‐mutant NSCLC (EGFR+ NSCLC): association between TP53 status and clinical outcome. Clin Lung Cancer. 2023;24:415–428. 37076395 10.1016/j.cllc.2023.02.010

[16] Schwaederlé M , Lazar V , Validire P , Hansson J , Lacroix L , Soria JC , et al. VEGF‐A expression correlates with TP53 mutations in non‐small cell lung cancer: implications for Antiangiogenesis therapy. Cancer Res. 2015;75:1187–1190. 25672981 10.1158/0008-5472.CAN-14-2305

[17] Li AM , Boichard A , Kurzrock R . Mutated TP53 is a marker of increased VEGF expression: analysis of 7,525 pan‐cancer tissues. Cancer Biol Ther. 2020;21:95–100. 31564192 10.1080/15384047.2019.1665956 PMC7012180

[18] Pfister NT , Fomin V , Regunath K , Zhou JY , Zhou W , Silwal‐Pandit L , et al. Mutant p53 cooperates with the SWI/SNF chromatin remodeling complex to regulate VEGFR2 in breast cancer cells. Genes Dev. 2015;29:1298–1315. 26080815 10.1101/gad.263202.115 PMC4495400

[19] Mazieres J , Drilon A , Lusque A , Mhanna L , Cortot AB , Mezquita L , et al. Immune checkpoint inhibitors for patients with advanced lung cancer and oncogenic driver alterations: results from the IMMUNOTARGET registry. Ann Oncol. 2019;30:1321–1328. 31125062 10.1093/annonc/mdz167 PMC7389252

